# Pre-surgical Brain Mapping: To Rest or Not to Rest?

**DOI:** 10.3389/fneur.2018.00520

**Published:** 2018-07-03

**Authors:** Cristina Rosazza, Domenico Zacà, Maria G. Bruzzone

**Affiliations:** ^1^Neuroradiology Unit, Fondazione IRCCS Istituto Neurologico “Carlo Besta,”, Milan, Italy; ^2^Center for Mind/Brain Sciences (CIMeC), University of Trento, Trento, Italy

**Keywords:** resting-state fMRI, task-based fMRI, preoperative setting, motor mapping, language mapping, functional connectivity

Preoperative mapping of brain functions is the most common clinical application of functional MRI (fMRI) (1). The pre-surgical localization of eloquent areas has a positive impact for maximizing the extent of resection while reducing intra-operative mapping time (2) and improving patient outcome (3). In the pre-surgical setting, the typical fMRI approach employs conventional tasks which require patients to execute simple tasks in the scanner (4–8). This task-based fMRI (tb-fMRI) approach is well established and widely used in clinical routine, but has limitations: patients must be able to perform the tasks appropriately, implementation has a cost, and trained personnel is needed to select the proper task and instruct the patient.

One straightforward alternative to task-based fMRI is resting-state fMRI (rs-fMRI). This technique allows the study of spontaneous, low-frequency fluctuations that occur throughout the brain and has been widely used to characterize the healthy brain and neurological diseases. With rs-fMRI it is possible to identify a number of networks, or components, which are strongly functionally connected at rest and highly reproducible across subjects and sessions (9–11). Some of them show similar topographies to tb-fMRI networks, that is regions activated during cognitive functions, visual or sensory-motor tasks (12, 13). Measures of functional connectivity allow the study of brain functional reorganization and neuronal changes associated with brain disease (14). Alterations of rs-fMRI networks have been identified in many neurological and psychiatric disorders, even in absence of structural modifications, and in some studies have been shown to correlate with disease progression and severity (15).

Rs-fMRI has been applied also in the preoperative setting to overcome the limitations of task-based fMRI, including the need of active patient participation (16). rs-fMRI has the additional advantage that acquisition is brief (~6–10 min) and can be easily managed by MRI technicians. Initial studies to validate the technique show good concordance with the gold standard intraoperative electro-cortical stimulation (ECS) (17–20). The most commonly identified networks for pre-surgical planning are the sensori-motor component, encompassing pre- and post-central cortex with the supplementary motor area, and the language network including Broca and Wernicke's areas, often corresponding to the fronto-parietal network (21–23).

## Open issues for presurgical rs-fMRI

Despite promising results, there remain open issues for the use of rs-fMRI in the preoperative setting. The methodology is the most critical issue: with rs-fMRI the acquisition is easy, but analyses are complex and have not been standardized yet. Multiple analysis techniques are available and, according to the method, different results can be obtained from the same dataset (11, 24, 25). Identification of the network is a critical step and can occur: (i) automatically or semi-automatically with Independent Component Analysis (ICA), a data-driven method commonly used: a network among a set of components is selected either visually (26) or through a spatial matching with respect to network templates (17, 27, 28, 29); (ii) manually, with a seed-based approach, where pre-defined region-of-interest (ROI)s or seeds are selected based on *a-priori* hypothesis; (iii) with alternative methods, such as machine learning approaches (18, 30, 31), cortical parcellating approach (19) or graph analyses (15).

Networks are not all as easily identifiable. The sensori-motor component is one of the lower-level networks with a known functional correlate (i.e., it corresponds to the sensori-motor system) which is robust and easy to identify at individual level (32). On the other hand, the identification of the language component is more challenging. Language is a “higher-order” function with substantial variability across subjects and several cognitive functions such as comprehension, production and perception (if a word is presented visually) are involved in language. For this reason, it remains to be determined whether a single language network can be observed. In addition, when language is mapped with ICA, the network showing the highest spatial overlap with the task-based map or known language areas is used (26, 28, 33, 34), while when it is mapped with the seed-based analysis, language ROIs of the left or right hemisphere or electrically stimulated points as seeds are used (17, 35, 36).

Language lateralization is also important in preoperative mapping, as surgical interventions in the dominant hemisphere are at higher risk of post-operative language deficits than interventions in the non-dominant hemisphere (37). rs-fMRI has been shown to provide results that are generally concordant with the Wada test for language dominance, similarly to tb-fMRI (33, 38). However, the degree of language lateralization measured with rs-fMRI varies considerably, depending on regions and methods used (35, 36, 39, 40). More studies assessing rs-fMRI lateralization with clinical, neuropsychological and post-surgical outcome evaluations are needed in order to determine which method (ICA or seed) provides the most reliable results (34, 41). At present, it is still unclear which method of analysis produces the most reliable results for mapping eloquent areas.

The second issue concerns the differences between rs-fMRI and tb-fMRI. The rs-fMRI map is different from the tb-fMRI one, as the two techniques are intrinsically different. As recently reported by Derks et al. (42), the two imaging methods measure different aspects of brain function. Tb-fMRI is related to the performance of a task or administration of a stimulus, and the resulting maps represent the brain areas involved. By contrast, rs-fMRI refers to brain's intrinsic activity, the degree of communication between areas and the resulting maps represent networks of synchronous BOLD activity (43). This may explain why the degree of overlap between the two techniques is not complete (30, 44, 45). In particular, the motor network observed at rest often covers a larger portion of the motor cortex compared to the more focal region identified with tb-fMRI (30, 44). Understanding whether a region is necessary for a function might be more difficult with rs-fMRI; in practice, future studies comparing rs-fMRI with ECS data will evaluate the accuracy of rs-fMRI results, using sensitivity and specificity measures (17–20). In other cases, the rs-fMRI motor network covers a smaller portion of the motor cortex when, for instance, a high number of components is selected with ICA, and the foot or mouth motor areas can be difficult to identify with ICA (44, 46).

**Figure 1:**
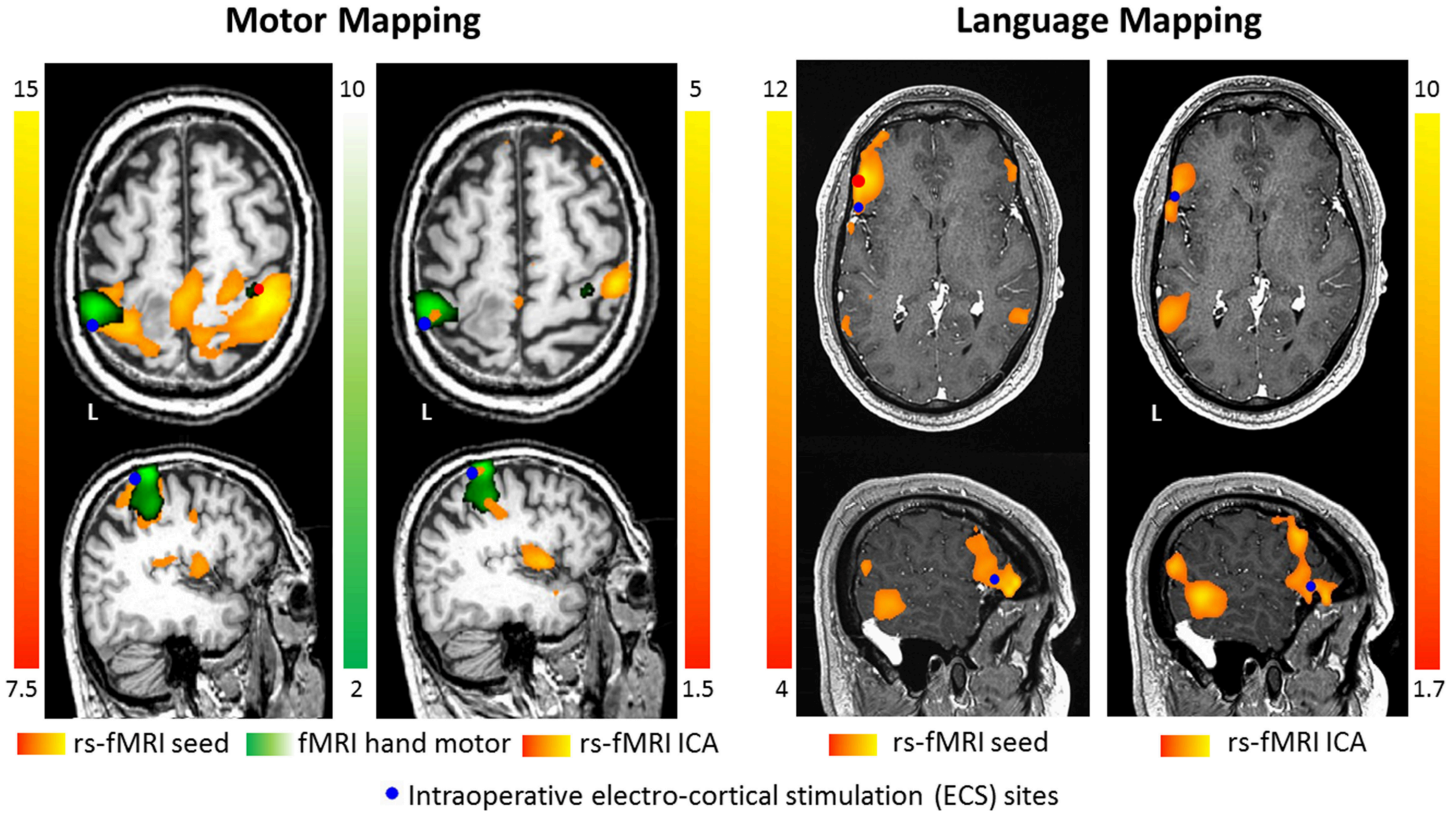
Motor mapping was performed in a surgical patient using rs-fMRI with motor seed (in red, placed in the healthy Rolandic cortex), rs-fMRI ICA, a finger tapping motor task and ECS data (patient 12 in Rosazza et al. (44)). Results showed that seed-based analysis, ICA and tb-fMRI, provide comparable but not equivalent results; nevertheless, the stimulation site (in blue) was included in each activation pattern. In another surgical patient language mapping was performed using rs-fMRI with language seed (in red, placed in the left inferior frontal gyrus), rs-fMRI ICA and ECS data (patient 6 in Zacà et al. (47)). Results showed that seed-based analysis and ICA provided similar but not equivalent results; however, stimulation site (in blue) was included in the pattern of correlated activity.

An example of motor and language mapping performed with rs-fMRI and ECS is illustrated in Figure 1. Motor mapping performed with rs-fMRI through a motor seed (placed in the healthy Rolandic cortex), provided a larger portion of sensori-motor areas compared to the finger tapping map (in green). In the same patient, ICA showed a different localization of the hand motor area compared to tb-fMRI and seed-based analysis. Nevertheless, the stimulation site which elicited motor responses was included in both rs-fMRI and tb-fMRI activations (patient 12 in Rosazza et al. (44)). In another patient, language mapping performed with rs-fMRI data through a language seed (placed in the left inferior frontal gyrus) and ICA, localized, to some extent, different language areas, even if the stimulation site was included in the activation pattern (patient 6 in Zacà et al. 47).

Among the factors that contribute to different rs-fMRI patterns, there is the placement of seeds when using the seed-based analysis, as seed size and location can bias the network, especially in lesioned brains. By contrast, when using ICA, networks can be combined or split, according to the small or big number of components used. In general, although ICA has ambiguity in choosing the number of components and identifying the components of interest, it is more explorative and less subjective than seed-based approach. Therefore, it may work better in patient populations, especially when large lesions may prevent the identification of reliable seeds in the eloquent cortex. In comparison with rs-fMRI, also tb-fMRI has some drawbacks, including: the choice of tasks used in particular for language mapping, the use of active vs. control conditions where it can be difficult to match the cognitive demands of the conditions and finally the use of two tasks that can be more time consuming than a single rs-fMRI sequence.

The third issue concerns the interpretation of rs-fMRI results. The pattern of correlated activity observed with rs-fMRI is not easy to interpret. With tb-fMRI, a map is associated to a behavior or cognitive function and this makes the results quite clear: in language mapping, for example, anterior regions are expected to be activated for production and so are posterior regions for comprehension; in addition, a differential involvement of anterior vs. posterior temporal regions is typically observed depending on the task (48, 49).

With rs-fMRI it is more difficult to understand the functional specificity of the map and interpret the results. The interpretation is even more difficult when rs-fMRI maps do not include expected areas, such as the paracentral lobule of the sensori-motor network. Most importantly, correlations of rs-fMRI pattern with cognitive or behavioral data have been established only occasionally. For instance, the rs-fMRI study by Otten et al. (50) has shown that in patients with brain tumors, motor weakness was associated with reduced connectivity of the sensori-motor network between inter-hemispheric motor areas. Further studies correlating measures of functional connectivity with clinical and cognitive data are necessary to understand the value of rs-fMRI in the clinical setting.

## Future development of rs-fMRI

Rs-fMRI has begun to make clinically meaningful contribution to the localization of eloquent areas. To make rs-fMRI standard of care for pre-surgical mapping, further developments in the methodological and theoretical setting are needed. From a methodological point of view, analyses must be reliable, quick and easily applicable in the preoperative routine for single patients. Initial steps have been made in this direction (51–53); however the procedure of analysis must be standardized, validated with ECS data and replicated in large population studies.

We can hypothesize that for motor mapping, the manual approach with the seed-based analysis will be better suited to localize the area of interest (foot, hand or mouth) with respect to the lesion. In fact, the seed-based analysis offers the flexibility necessary to explore the functional connectivity from different ROIs, even if it is more sensitive to the type of preprocessing performed. However, when the identification of motor seeds is confounded by pathology, ICA could provide a valid alternative approach (30, 53). In contrast, for language mapping, a semi-automatic approach with ICA could be better suited to assess lateralization and also localize language. ICA could be repeated setting a different number of components (e.g., 10, 20, 30.) and selecting the network that best matches a pre-defined template. This approach has been shown to provide reliable results because it takes into account both the variability of optimal number of components and localization of language areas across patients (52). When possible, the two methods of analysis should be applied jointly to obtain an independent confirmation of findings, similarly to the tb-fMRI procedure where two or more tasks are typically administered to map a function.

Considering the current limitations, we believe that, at present, tb-fMRI represents the first paradigm to choose for preoperative mapping of brain functions. Tb-fMRI is more robust with respect to noise, different tasks can be employed to map specific areas, analyses are quick, widely applied in the clinical routine, and maps are easy to interpret. When tb-fMRI cannot be used for clinical or logistic reasons, for instance because patients cannot perform the task or the stimulus delivery device is not available, then rs-fMRI will be used for the same purpose.

However, looking forward, we believe that, in the future, conceptual and methodological advances in neuroimaging techniques will allow a broader application of rs-fMRI functional connectivity in different neurological disease, including surgical practice. We are in fact shifting from a localizationist vision to a network-centric perspective, according to which the brain is organized into hierarchical, integrated, and interconnected large-scale networks, and neurological diseases are described as neuronal circuits dysfunctions (54–56). Network modeling of neuro-pathological conditions will be widely performed through connectivity analyses within and between networks, and results will be easily visualized to make rapid clinical decisions (15, 57). This is going to be accompanied by important theoretical advancements in clinical neuroscience: the functional relevance of rs-fMRI measures and their clinical correlates will be elucidated, together with a clearer definition of the areas of networks indicating eloquent cortex. rs-fMRI is going to be used also to localize glioma-related alterations and delineate the degree of tumor infiltration (14). From longitudinal studies it is going to be possible to understand network changes occurring throughout surgery and this will allow the development of personalized treatments (58). In this context, we can imagine that rs-fMRI will be used in the preoperative setting not only to map eloquent areas but also to get information about changes on neuronal circuits caused by lesions, and eventually it will provide the basis for a multi-network assessment for diagnosis, prognosis and treatment of single patients.

In conclusion, methodological and theoretical limitations currently prevent a routine use of rs-fMRI in the pre-surgical setting. However, there is a wider potential for this technique that it is likely to be realized in the future also for preoperative mapping.

## Author contributions

CR and MB suggested the idea of the work. CR and DZ drafted the manuscript. CR, DZ, and MB revised the manuscript. All authors have read and approved the manuscript in its final form.

### Conflict of interest statement

The authors declare that the research was conducted in the absence of any commercial or financial relationships that could be construed as a potential conflict of interest.
